# Research on equipment layout of multi-layer circular manufacturing cell based on NSGA III

**DOI:** 10.1371/journal.pone.0312364

**Published:** 2024-12-23

**Authors:** Yanlin Zhao

**Affiliations:** School of Intelligent Manufacturing, Panzhihua University, Panzhihua, Sichuan, P. R. China; University of Vienna: Universitat Wien, AUSTRIA

## Abstract

This paper investigates the layout optimization of multi-layer circular manufacturing cells (MCMC), a topic that has garnered limited attention compared to single-layer circular manufacturing cells (SCMC). With the continuous advancement of global intelligent manufacturing, MCMC has emerged as a viable solution, with several smart factories already implementing this model. Existing literature predominantly utilizes the NSGA II algorithm for SCMC layouts due to their relatively few objectives. However, the layout problem for MCMC encompasses a significantly larger number of objectives, rendering NSGA II inadequate. This study aims to fill this research gap by proposing an innovative approach using NSGA III, specifically designed for complex multi-objective optimization. A multi-dimensional target mathematical model for MCMC is established, facilitating the systematic examination of layout configurations. The methodology employs NSGA III to effectively tackle the challenges posed by MCMC layouts. To validate the effectiveness of NSGA III, empirical data from a smart factory in Zhejiang, China, is utilized. The findings demonstrate that NSGA III significantly outperforms traditional algorithms, yielding superior solutions for MCMC layout problems. This research not only challenges the conventional SCMC layout paradigm but also expands the options available for facility layouts in smart factories. Ultimately, it addresses the pressing engineering needs of smart factory construction and contributes valuable insights to the field of MCMC research, establishing a robust methodology for future studies.

## 1. Introduction

### Background and context

With the rise of intelligent factories, advanced concepts such as Cloud Manufacturing, the Internet of Manufacturing Things, and Cyber-physical production systems have emerged. These systems aim to integrate cutting-edge technologies into smart factory construction, facilitating the transformation and upgrading of production models [1,2]. As these models evolve, the demand for customized, small-batch production is increasing, which has led to the widespread adoption of Cellular Manufacturing Systems (CMS) in intelligent factory settings. CMS, originally developed from group technology, is designed to cater to multi-variety and small-batch production, making it well-suited for the diverse manufacturing needs of smart factories [3].

The engineering application of smart factories requires addressing the market’s diverse needs effectively. CMS organizes equipment into product families based on the similarity of production processes, promoting flexibility while maintaining the efficiency of larger assembly line operations. However, the shift towards intelligent logistics and uncrewed operations in smart factories has necessitated a reevaluation of manufacturing cell layouts, moving beyond traditional single-layer configurations to more complex Multi-layer Circular Manufacturing Cells (MCMC).

**Fig 1:**
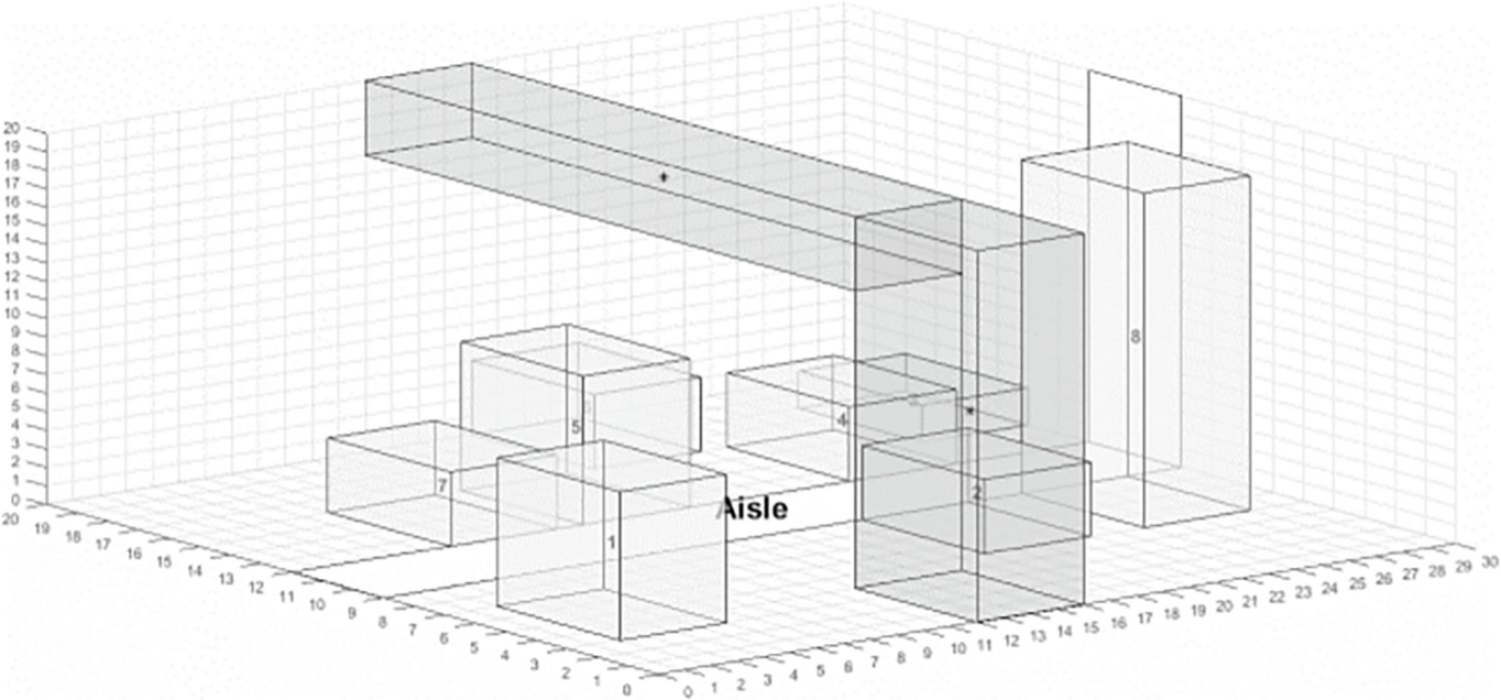
Layout of the primary 3D manufacturing cell facility [5].

MCMC layouts offer significant advantages when production capacity must expand in limited factory space or when production logistics costs are high. However, MCMC is still in the early stages of engineering application, facing challenges due to its complexity and the need for three-dimensional space considerations [4]. For example, the layout of the primary 3D manufacturing cell facility as shown in Fig 1. The MCMC layout problem is fundamentally a multi-objective combinatorial optimization problem, characterized by non-linear, discontinuous, and multi-constrained aspects that complicate problem-solving.

### Research gap and significance

Despite advancements in CMS research, there remains a notable gap regarding the environmental impacts associated with production distribution networks, particularly within the context of MCMC layouts [6]. Most existing studies have concentrated on operational efficiency and production performance, often neglecting the ecological consequences of manufacturing configurations. Addressing this gap is critical for developing sustainable practices that align with the growing emphasis on environmental responsibility in manufacturing.

This study aims to contribute to the existing body of knowledge by focusing on the environmental implications of MCMC layouts in smart factories [7]. By highlighting the necessity of integrating sustainability into facility design, this research seeks to provide valuable insights for both academics and practitioners, ultimately fostering a more sustainable approach to manufacturing.

### Objectives and scope

The primary objective of this research is to analyze the layout of Multi-layer Circular Manufacturing Cells (MCMC) and its environmental impacts within production distribution networks. The study will utilize a mixed-methods approach, employing both quantitative analysis and case studies to explore effective layout strategies that enhance sustainability. Specifically, the research will focus on developing a high-dimensional mathematical model to address the complexities of MCMC layouts, utilizing the NSGA III algorithm to optimize solutions.

### Structure of the paper

This paper is structured as follows:

Section 2 presents a literature review, discussing existing research on manufacturing cell layouts and their environmental impacts.

Section 3 outlines the mathematical problem description, detailing the key challenges associated with MCMC layouts.

Section 4 describes the model building and algorithm design, including the implementation of NSGA III.

Section 5 presents case applications to demonstrate the effectiveness of the proposed methodology.

Section 6 presents important managerial insights from this study for managers in smart manufacturing environments.

Section 7 concludes the paper with a summary of findings and recommendations for future research.

## 2. Literature review

### Related studies

Scholars’ research on manufacturing cell layout mainly includes layouts forms, mathematical models, and solution methods. Table 1 summarizes key studies in this area.

**Table 1:** Overview of studies on manufacturing cell layouts.

Study	Layout Type	Focus	Key Findings
Yu [8]	Linear	Layout of linear manufacturing cells	Analyzed efficiency in material handling.
Liu [9]	Linear	Layout optimization	Proposed enhancements for workflow efficiency.
Li [10]	Linear	Material handling costs	Focused on minimizing total costs in material movement.
Bao [11]	U-shaped	U-shaped manufacturing cells	Found advantages in space utilization and flexibility.
Bányai [12]	U-shaped	Layout efficiency	Highlighted performance improvements in U-shaped configurations.
Zheng [13]	Circular	Ring-shaped robot cells	Concluded suitability for robotic manufacturing.
Peng [14]	Circular	Ring-shaped manufacturing cells	Emphasized benefits in robotic applications.
Zhao [15]	Integrated	Multi-layout forms	Proposed integrated layouts combining different forms.
Mariem [5]	3D	Multi-layer equipment layout	Developed a mathematical model transitioning from 2D to 3D.

The most commonly used forms of cell layout are linear, U-shaped, and circular. For example, Yu [8], Liu [9], Li [10], and others have studied the layout of linear manufacturing cells. Bao [11] and Bányai [12] have researched U-shaped manufacturing cells. Zheng [13] and Peng Y [14] conducted studies on ring-shaped robot cells and concluded that these cells are suitable for robotic manufacturing. Scholars’ research on manufacturing cells mainly focuses on single-layer layouts, with few documents addressing multi-layer layouts. Zhao [15] proposed an integrated layout with multiple forms. Mariem [5] proposed a 3D equipment layout, constructed a mathematical model for multi-layer equipment arrangements in workshops, and solved it. The work of these two scholars has shifted the traditional 2D equipment layout into the 3D era.

The manufacturing cell layout problem has evolved from an initial single-objective optimization problem focused on minimizing the total logistics flow to a dual-objective optimization problem that minimizes both the total logistics flow and total floor area, and now to a multi-objective optimization problem that considers various factors. Early research by Li [16] addressed the single-objective problem of minimizing the total cost of material handling in manufacturing cell layouts. Arkat [17] constructed a bi-objective optimization mathematical model that addresses total transportation cost and completion time. Mohtashami [18], Golmohammadi [19], and Farboodi [20] improved the fast non-dominated sorting genetic algorithm and the particle swarm algorithm to develop a new algorithm for solving the multi-objective problem of manufacturing cell layout, achieving good results.

The manufacturing cell equipment layout problem has shifted from early single-objective research to current multi-objective studies. Various optimization algorithms are available for multi-objective problems. Initially, precise algorithms dominated, but now intelligent algorithms are more prevalent. Professor Kalyanmoy Deb [21] categorized multi-objective algorithms into two types. The first type converts multi-objective problems into single-objective problems using preference selection factors. The second type retains the attributes of the original objective functions and employs a specific selection strategy to choose individuals with excellent characteristics for iteration. The first type of algorithm alters the original objective function attributes, meaning that the strategic choice of preference selection factors directly affects the solution results, which complicates their use. In contrast, the second type does not alter the original objective function attributes and focuses on multi-objective excellence selection.

In 1994, Kalyanmoy Deb and Nidamarthi Srinivas proposed the non-dominated sorting method [22]. By retaining multi-objective attributes, excellent individuals are selected for iteration through non-dominated sorting, opening up a research path for non-dominated sorting evolutionary algorithms [23]. The first generation of the non-dominated sorting genetic algorithm (NSGA) faced issues with slow search speeds and excessive computation. To address these problems, Professor Deb proposed the fast non-dominated sorting genetic algorithm (NSGA II) in 2000. Since its launch, NSGA II has been favored by scholars and engineers and widely applied, including by Shahab Safaei [24], Peiman Ghasemi [25], and A Babaeinesami [26] in solving facility layout problems. NSGA II has achieved good results [27–29]. In multi-objective optimization applications, NSGA II excels in solving problems with three or fewer objectives. However, as the number of solution targets increases, its performance declines. Therefore, in 2014, Professor Deb proposed the non-dominated sorting genetic algorithm III (NSGA III), based on reference points. Since its introduction, NSGA III has been widely used in engineering applications and academic research, yielding promising results. However, in the context of equipment layout, NSGA II remains the primary solution both domestically and internationally, with few scholars applying NSGA III to the equipment layout of manufacturing cells.

### Research gap analysis and contributions

Despite significant advancements in manufacturing cell layout research, most studies primarily focus on single-layer layouts, leaving a substantial gap in the exploration of multi-layer circular manufacturing cells (MCMC). While algorithms like NSGA II have demonstrated effectiveness in solving single-layer manufacturing cell layout problems with a limited number of objectives, they falter when applied to MCMC, where the complexity increases due to a higher number of targets and equipment arrangements. This limitation highlights the necessity for more robust methodologies tailored to the multi-dimensional nature of MCMC.

Furthermore, existing literature reveals that while some scholars, such as Zhao and Mariem, have ventured into integrated and 3D layouts, their approaches often do not address the unique challenges posed by multi-layer configurations, particularly in dynamic manufacturing environments. The lack of comprehensive models and algorithms for MCMC not only constrains practical applications in modern smart factories but also hinders academic discourse in this emerging area.

This paper aims to bridge this research gap by constructing a novel mathematical model specifically for MCMC layout problems and introducing NSGA III as a solution method. NSGA III is better equipped to handle the high-dimensional target optimization required for MCMC, leveraging its ability to maintain the integrity of multiple objectives during the optimization process. Through case studies and empirical validation, this research not only demonstrates the effectiveness of NSGA III in addressing MCMC layout challenges but also provides valuable insights into the design and implementation of multi-layer manufacturing systems. By filling this critical gap, the study contributes to both theoretical frameworks and practical applications, offering a foundation for future research in the evolving landscape of intelligent manufacturing.

## 3. Problem description

MCMC mainly includes processing equipment, conveyor belts, elevators, processed products, guardrails, stairs, and multi-layer platforms. Among these, the layout of processing equipment is the focus of this article, so "equipment" in this context refers specifically to the equipment that processes products.

**Fig 2:**
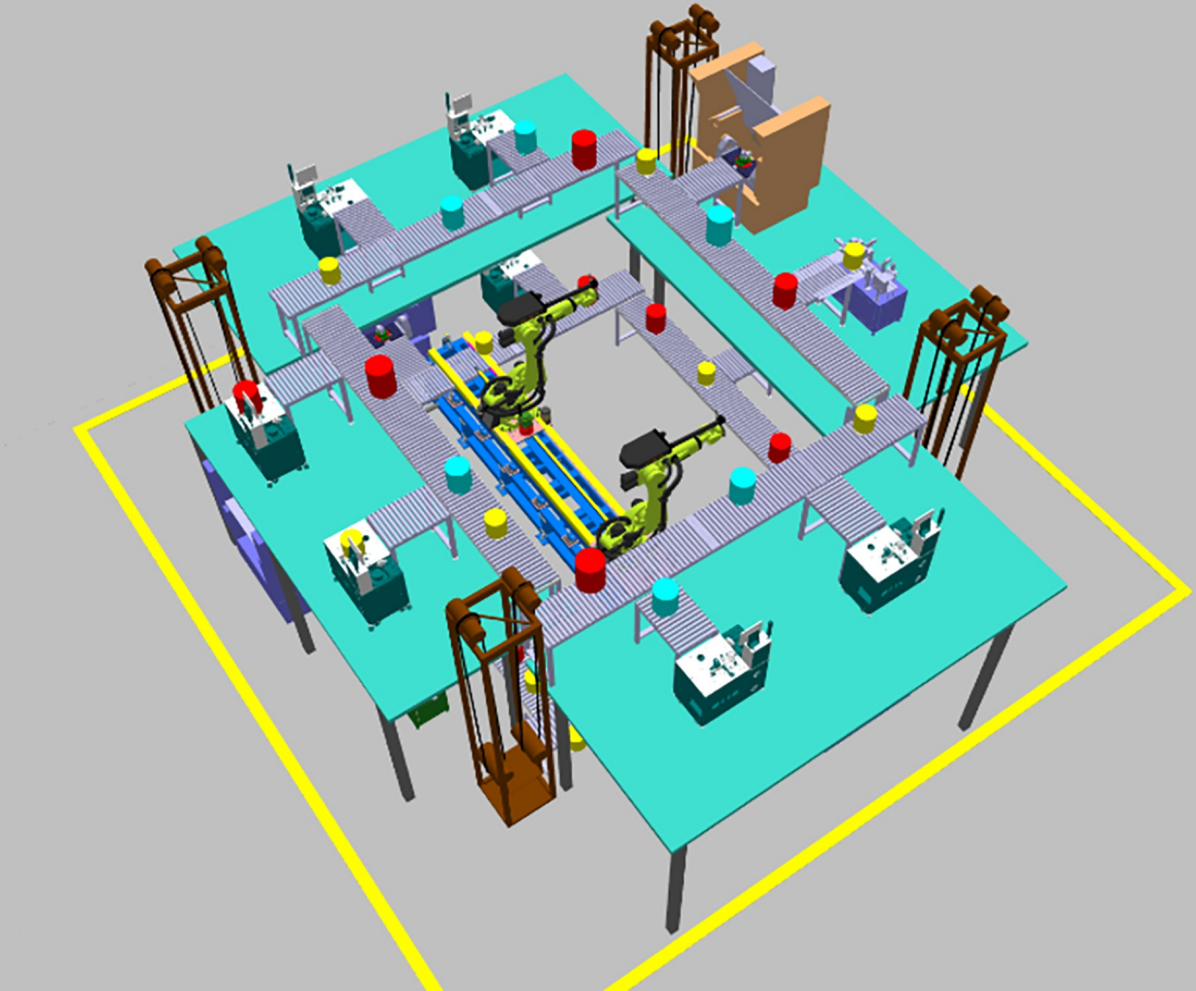
MCMC three-dimensional view.

In the MCMC layout, the starting point and endpoint of logistics are the same, forming a logistics loop; thus, it is called a Multi-layer Circular Manufacturing Cell (MCMC). MCMC equipment is arranged across four planes, with each plane containing at least one level of equipment. The conveyor belts on each floor form a circular loop, and a material-handling robot is positioned in the center of this loop. A three-dimensional diagram of the MCMC layout is shown in Fig 2.

**Fig 3:**
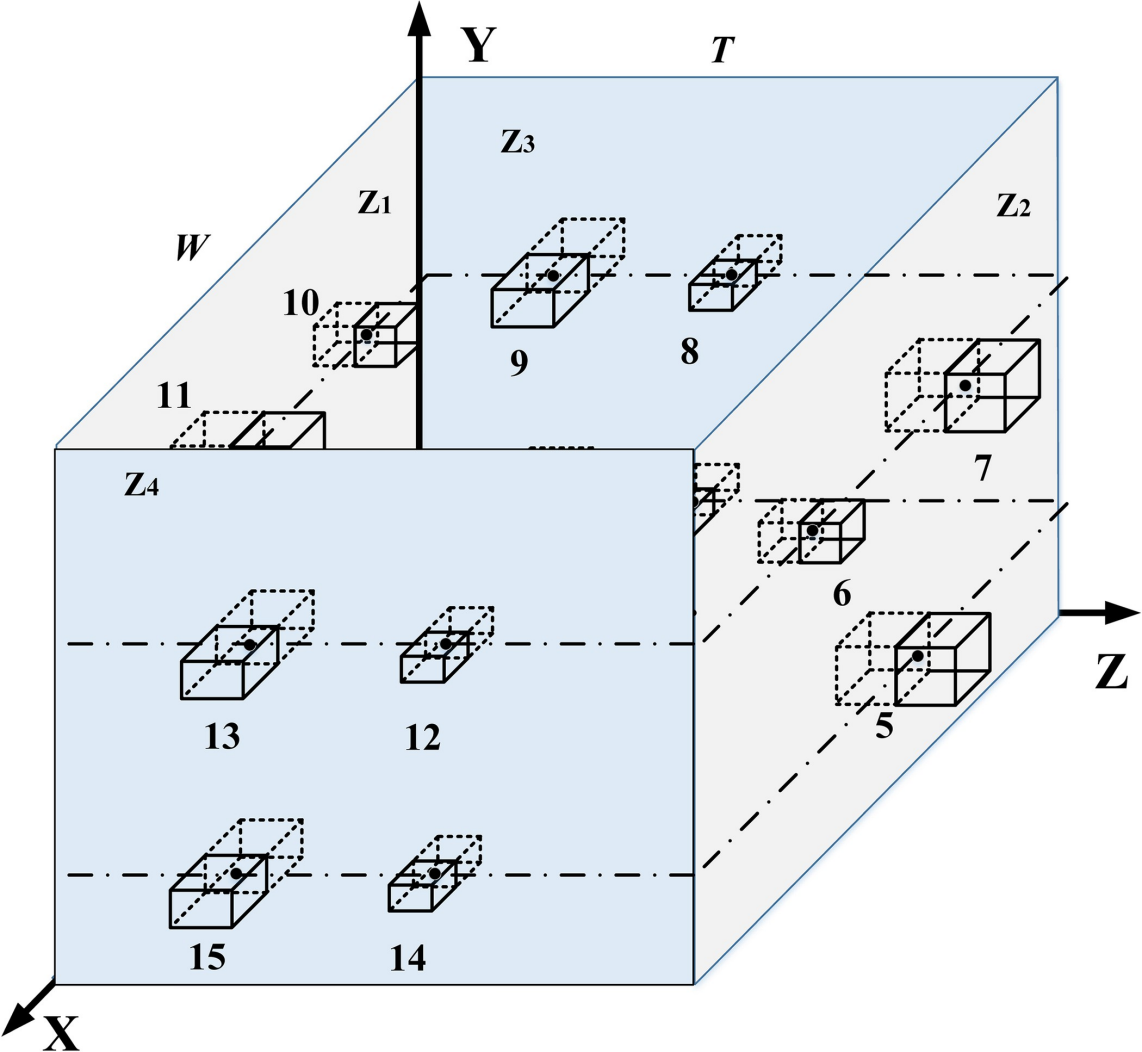
MCMC abstract layout diagram.

We abstract Fig 2 and construct an XYZ rectangular coordinate system. The XOY plane corresponds to the Z1 plane, while the plane parallel to the XOY plane is the Z2 plane. The YOZ plane represents the Z3 plane, and the plane parallel to the YOZ plane is the Z4 plane. Multi-layer equipment is placed on the four planes—Z1, Z2, Z3, and Z4—respectively, with the centerline of the equipment forming a loop, as shown in Fig 3.

**Fig 4:**
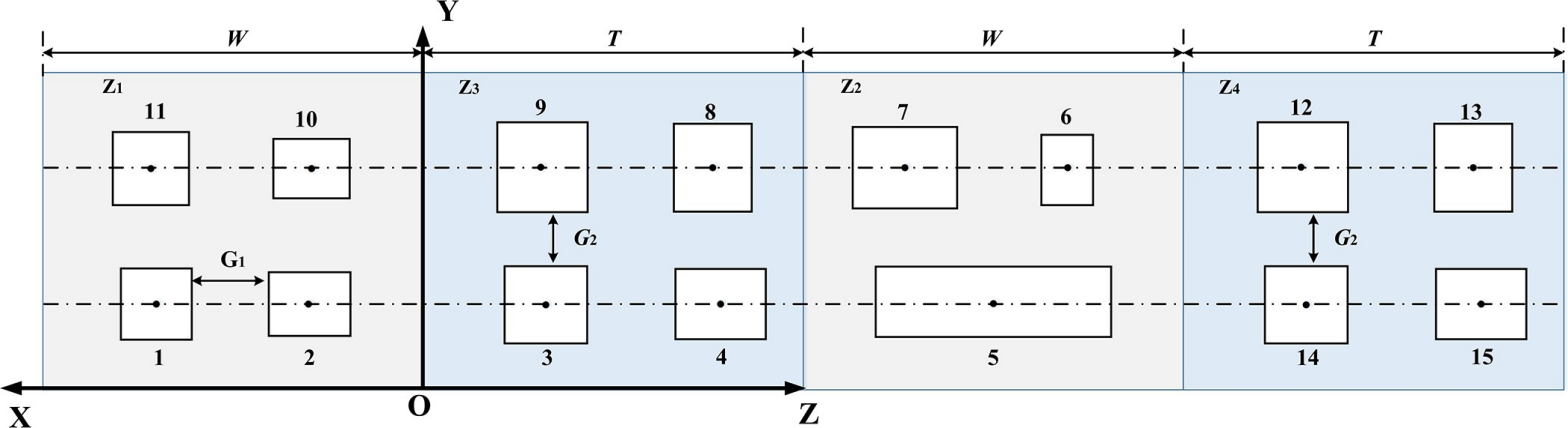
MCMC plane plan.

We use Z3 as the datum plane and expand the MCMC at the intersection of Z4 and Z2. The equipment on the expanded floor plan is sized according to its projection onto each plane, as shown in Fig 4.

MCMC is a layout form of multi-layer manufacturing cells and includes single-layer manufacturing cell layouts. The single-layer manufacturing cell is a specific case where the number of layers *r* = 1 in the multi-layer manufacturing cell. Therefore, the layout of the multi-layer manufacturing cell and the layout of the single-layer manufacturing cell have an inclusive relationship, with the latter being a subset of the former.

## 4. Multi-objective model building

Currently, MCMC is still in the budding stage of engineering applications and is mainly used in smart factories with a high degree of automation, in the layout of local multi-layer manufacturing cells, and in robotic manufacturing cells. Based on company visits and exchanges, the engineering application requirements for MCMC are primarily focused on the following points:

- The planned land area of the factory is limited.
- Land is expensive.
- The factory layout needs to be compact.
- Market demand is variable, requiring increased production flexibility.
- A significant amount of equipment needs to be added to the original site.
- Material handling costs remain high.
- There is a demonstrative effect of smart factories.

In addition to these needs, MCMC can reduce work-in-progress inventory, improve the production balance rate to lower manufacturing costs, shorten manufacturing time to enhance customer service levels, and allow for quick adjustments in equipment to respond to market changes.

We summarize these requirements into key issues such as occupied space, distance for moving materials, production line balance, stability, and non-logistics relationships. For instance, occupied space corresponds to factors like expensive land, limited planning area, and compact production lines. The distance for moving materials relates to the high cost of material handling. Production line balance is associated with the flexibility of the production line and the number of products in process. The relationship between stability and non-logistics is mainly considered from the perspective of equipment attributes and product process requirements. Therefore, MCMC takes into account factors such as occupied space, distance for transporting materials, production line balance (cell loss time), stability, and non-logistics relationships, and builds a multi-objective model of MCMC based on these factors.

### Material handling objective

In a smart factory, the equipment for processing products is highly intelligent, and the material handling equipment is automated. Consequently, the material handling method primarily involves unitized handling equipment, which can manage one or more cell products at a time. Let *V*_*p*_ represent the processing batch size of product *p*, and *B*_*p*_ represent the handling batch size of product *p*. The material handling volume between equipment *i* and equipment *j* is given by *ceil*[*V*_*p*_/*B*_*p*_], where *ceil*[] denotes the ceiling function (rounding up to the nearest integer). Since this study focuses on smart factories, transportation relies on automated handling equipment, and weight does not significantly impact machine handling. Therefore, the logistics handling volume does not account for weight factors, but considers distance, handling volume, and handling times. In factories with a high degree of automation, the handling distance approximates the Manhattan distance; thus, this article uses Manhattan distance as the logistics handling distance. The material handling objective function is represented in Eq (1).


$$\min\;D=\sum_{i=1}^{M}\sum_{j=1}^{M}\sum_{p=1}^{P}ceil[V_{p}/B_{p}](\left|x_{i}-x_{j}|+\left|y_{i}-y_{j}\right|+|z_{i}-z_{j}\right|)$$ (1)

- *D* represents the total amount of material handling for processing tasks.
- *i* and *j* represent the device numbers.
- *p* represents the product category number being processed.
- *P* represents the total number of product types being processed.
- *M* represents the total number of equipment units.
- *V*_*p*_ represents the total quantity of product *p* to be processed.
- *B*_*p*_ represents the quantity of product *p* transported at one time.
- *x*_*i*_, *y*_*i*_, *z*_*i*_ represent the center coordinates of device *i*.
- *ceil*[] denotes rounding up to the nearest integer

**Fig 5:**
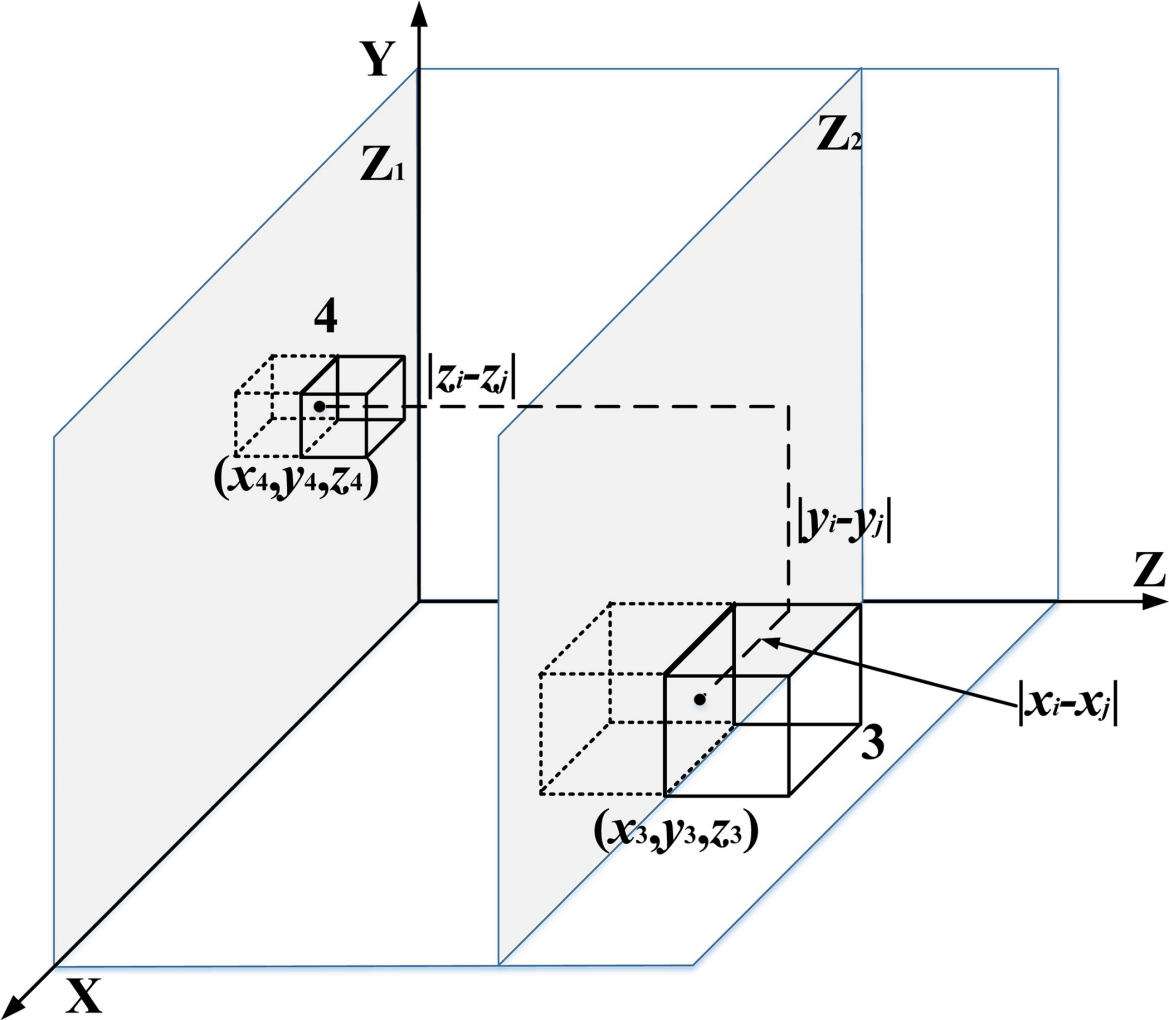
Different plane moving paths.

**Fig 6:**
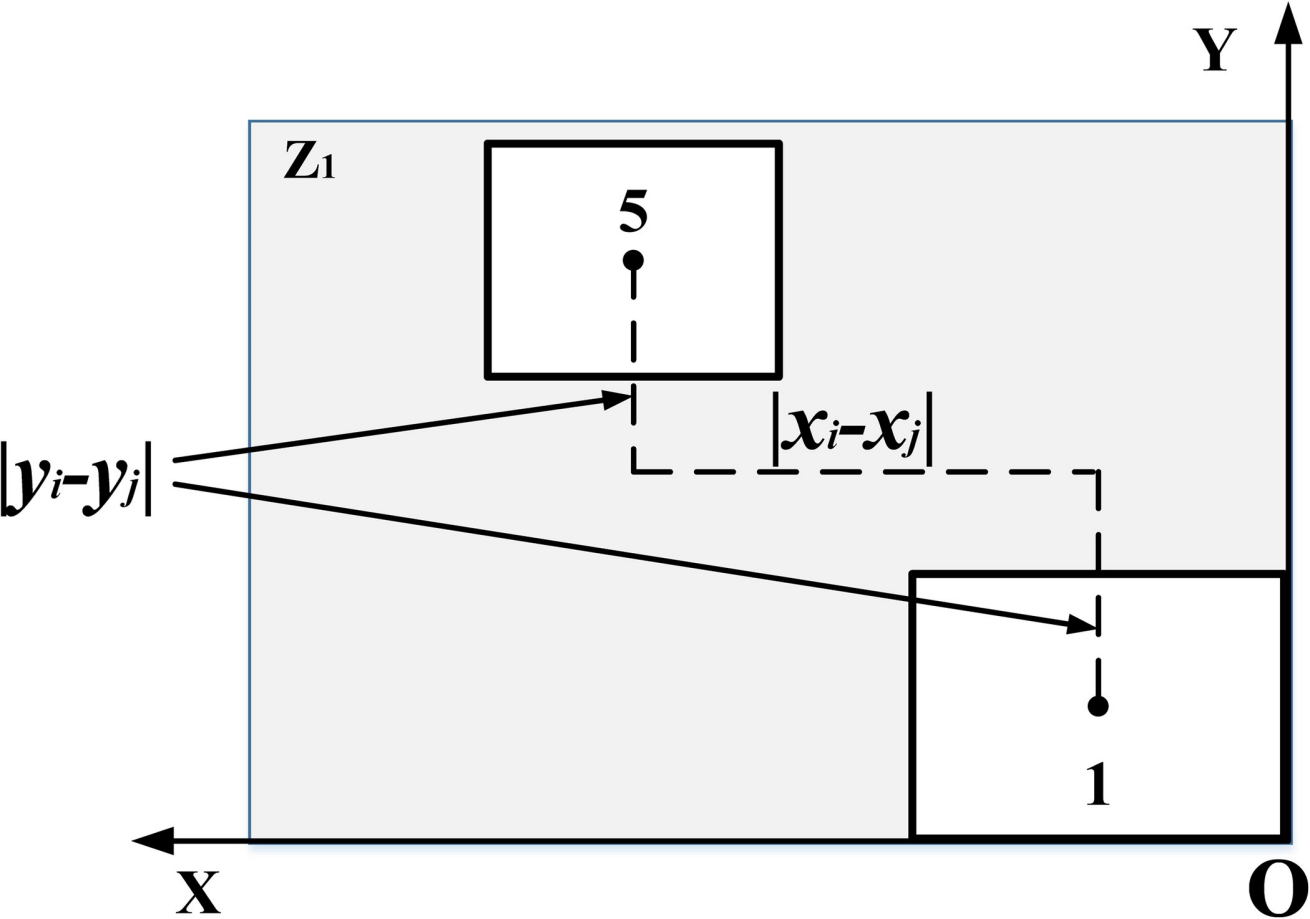
Moving path on the same plane.

The distance between equipment *i* and equipment *j* is measured based on the center points of the equipment. Since current intelligent logistics handling systems, such as robotic arms or conveyor belts, primarily operate with right-angle turns in the three directions of X, Y, and Z, the Manhattan distance is selected for this analysis. As shown in Fig 5, the movement path is between the two planes Z1 and Z2, while Fig 6 illustrates the movement path within the same plane.

### Cell volume objective

The volume objective function of MCMC must consider three elements: the cell’s length, width, and height. When these three dimensions are minimized, the cell volume is also minimized. Therefore, in constructing the objective function, it is necessary to develop separate objective functions for each of the three elements while retaining the resulting output values for length, width, and height. The cell volume objective function is formulated in Eqs (2) to (5).

*(1)*Length objective function;

$$\begin{array}{l} \min\;C=\max\left\{x_{i}+\frac{\left(l_{i}f_{i}+w_{i}(1-f_{i})\right)}{2}\right\}\\ \;-\min\left\{x_{i}-\frac{\left(l_{i}f_{i}+w_{i}(1-f_{i})\right)}{2}\right\} \end{array}$$ (2)

- *C* represents the length of the cell.
- *x*_*i*_ represents the *x* value representing the center point of device *i*.
- *l*_*i*_ and *w*_*i*_ represent the dimensions projected onto the *X*-axis and *Z*-axis when device *i* is first laid out.
- *f*_*i*_ indicates whether the device has a condition for horizontal rotation.

The cell length objective function is the set of the sum of the *x* value of the center coordinate of the device and half of the size of the device projected onto the *X* axis, and then find the difference between the maximum value and the minimum value in the set to be the cell length. When arranging cell equipment, some equipment can be arranged in one direction in the XOZ plane, so *f*_*i*_ is introduced to determine whether the equipment is rotated. If the equipment does not have the conditions for turning, then the value ratio of fi is 1. If the equipment has the conditions for turning, then *f*_*i*_ can be 0 or 1.

*(2)*Width objective function;

$$\begin{array}{l} \min\;K=\max\left\{z_{i}+\frac{\left(l_{i}(1-f_{i})+w_{i}f_{i}\right)}{2}\right\}\\ \;-\min\left\{z_{i}-\frac{\left(l_{i}(1-f_{i})+w_{i}f_{i}\right)}{2}\right\} \end{array}$$ (3)

*K* represents the width of the cell.

*z*_*i*_ represents the z value of the center point of device *i*.

*l*_*i*_ and *w*_*i*_ means that when device *i* is first laid out, it is projected onto the *X*-axis and *Z*-axis.

*f*_*i*_ represents the same value as fi in the length objective function.

The cell width objective function is the set of the sum of the z value of the center coordinate of the device and half of the size of the device projected onto the *Z* axis, and then find the difference between the maximum value and the minimum value in the set to be the cell width. When arranging cell equipment, some equipment can be arranged in one direction in the XOZ plane, so fi is introduced to determine whether the facilities are arranged in a direction. If the equipment does not have the conditions for turning, then the value ratio of *f*_*i*_ is 1. If the equipment has the conditions for turning, then *f*_*i*_ can be 0 or 1.

*(3)*Height objective function;

$$\min\;H=\max\{y_{i}+\frac{h_{i}}{2}\}$$ (4)

- *H* represents the height of the cell.
- *y*_*i*_ represents the y value representing the center point of device *i*.
- *h*_*i*_ represents the height of device *i*.

The cell height objective function is a set of the sum of the y value of the center coordinate of the equipment and half of the size of the facility projected onto the Y axis, and then finding the maximum value in the set is the cell height.

*(4)*Volume objective function;

minV=CKH (5)

- *V* represents the volume of the cell.
- *C* represents the length of the cell.
- *K* represents the width of the cell.
- *H* represents the height of the cell.

Find the minimum value of *C*, *K*, *H*, which is also the minimum value of cell height *V*. Therefore, the three objective functions of length, width, and height can be synthesized into a volume objective function, but the calculation process must retain the values of *C*, *K*, and *H* at the minimum volume, because there are multiple values of *C*, *K*, and *H* in the same volume. Engineering applications require the selection of appropriate C, *K*, and *H* values based on actual needs.

### Cell loss time objective

The production balance of MCMC directly affects the efficiency of the cell, the number of work-in-progress, and the smoothness of the cell. MCMC’s balance indicators include balance rate, loss rate, and loss time. These three indicators can all indicate the balance of the manufacturing cell. This paper uses lost time as the production balance index of MCMC.

In the material handling target model, the smaller the total handling distance is, the better. However, this goal may not necessarily achieve the shortest manufacturing loss time of the cell. In the production process of a product, the equipment processing time can be regarded as a fixed value. The transportation time between equipment in the product process route changes with the different cell equipment layouts. For the bottleneck process, arrival too fast or too slow will cause time loss and increase the number of work-in-process in the cell. Therefore, the production line balance needs to be considered in the cell to construct an objective function that minimizes the manufacturing loss time of the cell.

**Fig 7:**
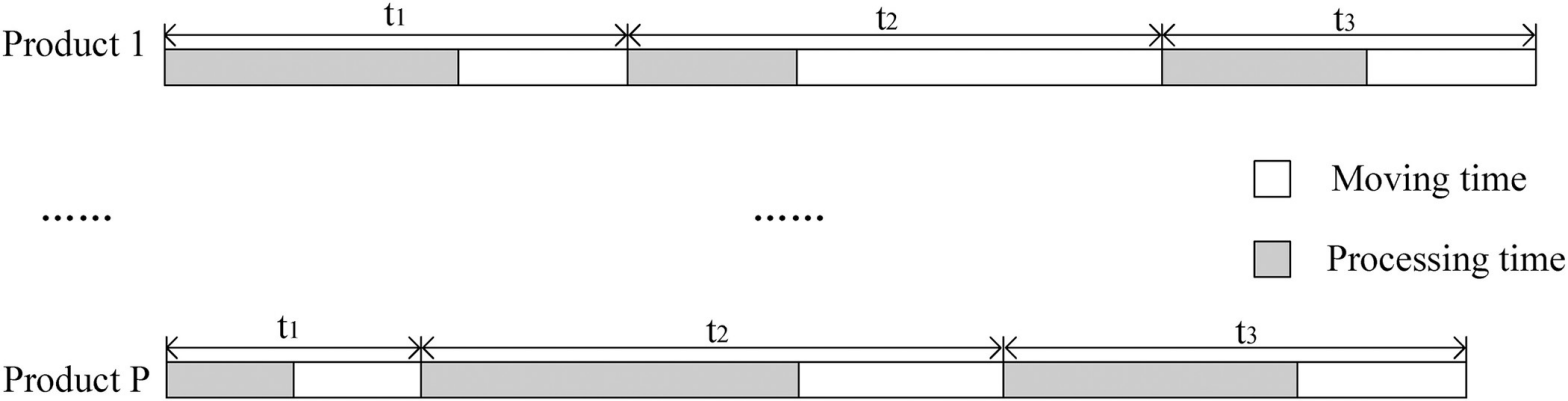
Time loss for product 1.

As shown in the time axis diagram of product 1 in Fig 7, the bottleneck process is t_2_, and we need to satisfy min(|t_1_-t_2_|+|t_2_-t_3_|), and so on to all products, to construct the cell manufacturing loss time objective function Formula (6) and Eq (7).


$$T_{p}=[(t_{i}^{p}+\delta (\left|x_{i}-x_{j}|+|y_{i}-y_{j}|+|z_{i}-z_{j}\right|))f_{ij}^{p}]$$ (6)

$$\begin{array}{l} \min\;T=ceil[V_{p}/B_{p}]\\ \;\sum_{p=1}^{P}\left(\max(T_{p})\mathrm{length}(T_{p})-\mathrm{sum}(T_{p})\right) \end{array}$$ (7)

- *T* table shows the total lost time in cell manufacturing.
- *T*_*p*_ represents the process path time set of *p* type products, including equipment processing time and handling time, such as the set of product 1 in Fig 7 [t_**1**_, t_**2**_, t_**3**_].
- $t_{i}^{p}$ represents the processing time of type p products at facility *i*.
- δ represents the conversion factor between distance and time.
- $f_{ij}^{p}$ is a 0–1 variable, indicating that the process path of type *p* products passes through equipment *i* and *j*.
- *p* and *P* represent the total number of types of products and processed products respectively.
- length() and sum() respectively represent the total number of elements in the collection and the sum of all element values in the collection.

### Cell stability objective

The layout of MCMC is a high-rise cell, and the stability of the cell will affect the cell’s infrastructure cost, operational safety, and ease of installation. The more stable the cell, the heavier the equipment, the lower the center of gravity, the better, so the objective function is constructed as shown in Eq 8.


$$\min\;B=\sum_{i=1}^{M}y_{i}W_{i}$$ (8)

- *B* represents the cell stability objective function. The smaller *B* is, the more stable the cell is.
- *y*_*i*_ represents the center point height of the device.
- W_**i**_ is the weight of device *i* (unit: kg).

### Non-logistics relationship objective

In equipment layout problems, non-logistics relationships are one of the essential objectives to consider. Some equipment attributes do not want to be close, and some equipment attributes can be close. For example, the positional relationship between high-temperature equipment and flammable equipment is undesirable. Therefore, non-logistics relationships are one of the factors that must be considered in equipment layout. The non-logistics relationship consists of the adjacency value and adjacency factor of the non-logistics relationship between devices. Before cell layout, the adjacency value between devices has been obtained through qualitative analysis. The larger the score, the closer the device is expected to be, and the closer the device is expected to be.) Referring to the research results of other scholars, the adjacency value and adjacency factor are set as shown in Tables 2 and 3.

**Table 2:** Non-logistics relationship adjacency value between devices.

Adjacency level	Symbol	Adjacency value(*a*_*ij*_)
Absolutely necessary	A	5
Very important	E	4
Important	I	3
Generally	O	2
Unimportant	U	1

**Table 3:** Non-logistics relationship adjacency factor.

Device distance(*b*_*ij*_)	adjacency factor (*d*_*ij*_)
$\left(d_{ij}^{\max}/5,2d_{ij}^{\max}/5\right]$	4
$\left(2d_{ij}^{\max}/5,3d_{ij}^{\max}/5\right]$	3
$\left(3d_{ij}^{\max}/5,4d_{ij}^{\max}/5\right]$	2
$\left(4d_{ij}^{\max}/5,d_{ij}^{\max}\right]$	1

The non-logistics relationship objective function is shown in Formula (8) and Formula (10).


$$d_{ij}=|x_{i}-x_{j}|+|y_{i}-y_{j}|+|z_{i}-z_{j}|$$ (9)

$$\min\;E=\sum_{i=1}^{M-1}\sum_{j=i+1}^{M}\frac{1}{a_{ij}b_{ij}}$$ (10)

- *d*_*ij*_ represents the Manhattan distance from device *i* to device j.
- *E* represents the total value of cell non-logistics relationships.
- *a*_*ij*_ represents the adjacency value from device *i* to device *j*.
- *b*_*ij*_ represents the adjacency factor from device *i* to device *j*.
- *M* represents the total number of devices.

### Constraint analysis

The constraints of MCMC mainly include circular layout constraint, boundary constraint, equipment non-interference constraint (non-overlapping constraint), same-layer equipment centerline constraint, equipment position uniqueness constraint and line wrapping constraint.

1. Circular layout constraint
  Equipments of MCMC are arranged on the Z1, Z2, Z3, and Z4 planes, f represents the 0–1 decision variable, and the construction constraints are shown in Eq (11).
$$\left\{ \begin{array}{l} f_{i}^{z_{1}}+f_{i}^{z_{2}}+f_{i}^{z_{3}}+f_{i}^{z_{4}}=1\\ \sum_{i=1}f_{i}^{z_{1}}\geq 1\\ \sum_{i=1}f_{i}^{z_{2}}\geq 1\\ \sum_{i=1}f_{i}^{z_{3}}\geq 1\\ \sum_{i=1}f_{i}^{z_{4}}\geq 1 \end{array}\right.$$ (11)
  $f_{i}^{z_{1}}+f_{i}^{z_{2}}+f_{i}^{z_{3}}+f_{i}^{z_{4}}=1$ indicates that the device must be in one of Z1, Z2, Z3, and Z4. $\sum_{i=1}f_{i}^{z_{1}}\geq 1,\sum_{i=1}f_{i}^{z_{2}}\geq 1,\sum_{i=1}f_{i}^{z_{3}}\geq 1,\sum_{i=1}f_{i}^{z_{4}}\geq 1$ indicates that at least one device must be arranged on each plane of Z1, Z2, Z3, and Z4.
2. Boundary constraint
  This constraint means that any device cannot exceed the boundaries of the cell. The constraints are shown in Eq (12).
$$\left\{ \begin{array}{l} x_{i}+\frac{\left(l_{i}f_{i}+w_{i}(1-f_{i})\right)}{2}\leq C_{g}\\ z_{i}+\frac{\left(l_{i}(1-f_{i})+w_{i}f_{i}\right)}{2}\leq K_{g}\\ y_{i}+\frac{h_{i}}{2}\leq H_{g} \end{array}\;\forall i\right.$$ (12)
  *K*_*g*_ represents the length boundary constraint of the cell in the *X*-axis direction.
  *C*_*g*_ represents the width boundary constraint in the *Z*-axis direction of the cell.
  *H*_*g*_ represents the height constraint in the *Y*-axis direction of the cell.
  (*x*_*i*_,*y*_*i*_,*z*_*i*_) represents the coordinate value of any device *i*.
  (*l*_*i*_, *w*_*i*_, *h*_*i*_) represents the size of any device *i*.
3. Equipment non-interference constraint
  The device non-interference constraint includes devices not overlapping and satisfying the device minimum spacing constraint. The constraints are shown in Eq (13).
$$\left\{ \begin{array}{l} |x_{i}^{r}-x_{i+1}^{r}|\geq \frac{\left(w_{i}f_{i}+l_{i}(1-f_{i}))+(w_{i+1}f_{i+1}+l_{i+1}(1-f_{i+1})\right)}{2}\\ \;+G_{1}\;\forall i,r\;\exists i+1\\ |y_{i}^{r}-y_{j}^{r+1}|\geq \frac{h_{i}+h_{j}}{2}+G_{2}\;\forall i,j,r\;\exists r+1\\ |z_{i}^{'}-z_{j}^{"}|\geq \frac{\left(l_{i}(1-f_{i})+w_{i}f_{i})+(l_{j}(1-f_{j})+w_{j}f_{j}\right)}{2}\\ \;+G_{3}\;\forall i,j \end{array}\right.$$ (13)
  - $x_{i}^{r}$ and $x_{i+1}^{r}$ respectively represent the coordinate *x* value when adjacent device *i* and device *i*+1 are both on the same layer *r*, then $\left|x_{i}^{r}-x_{i+1}^{r}\right|$ represents the distance limit between adjacent device coordinates on the same layer;
  - $y_{i}^{r}$ and $y_{j}^{r+1}$ respectively represent the coordinate y values of device i and device j when they are in layer r and layer r+1 respectively, then $\left|y_{i}^{r}-y_{j}^{r+1}\right|$ represents the distance limit between device coordinates when they are on different and adjacent layers;
  - $z_{i}^{'}$ and $z_{j}^{"}$ MM respectively indicate the distance limit in the Z-axis direction when equipment i and equipment j are located directly opposite, such as (Z1, Z2) or (Z3, Z4);
  - G1, G2, and G3 are respectively given constant values, indicating the minimum distance limit between device surfaces.
  - Same-layer equipment centerline constraint;
  In engineering applications, the height parameters of conveyor belts on the same layer are consistent. After the equipment processes the product, it is transported to the conveyor belt for material transfer. Therefore, this article assumes that the logistics delivery of equipment is the facility center, and the equipment center point is on the same horizontal line. The construction constraints are shown in Eq (14).
$$y_{i}^{r}=y_{j}^{r}$$ (14)
  $y_{i}^{r}$ and $y_{j}^{r}$ bb represent the coordinate y values of device *i* and device *j* on layer *r* respectively.
4. Equipment position uniqueness constraint;
  The uniqueness of equipment location means that the equipment can and can only be arranged in a certain location of the cell, and cannot be arranged in multiple locations at the same time. This is equivalent to the fact that the Manhattan distance between any two devices cannot be 0. The constraints are shown in Eq (15).
$$|x_{i}-x_{j}|+|y_{i}-y_{j}|+|z_{i}-z_{j}|>0\;i\neq j\;\forall i,j$$ (15)
  (*x*_*i*_, *y*_*i*_, *z*_*i*_), (*x*_*j*_, *y*_*j*_, *z*_*j*_) respectively represent the coordinate values of arbitrary equipment *i* and equipment *j*. If *i*≠*j* represents two different arbitrary equipment positions, then the difference between the coordinates of any different equipment in the cell is not 0, which indicates the uniqueness of all equipment arrangements.
5. Line wrapping constraint;
  The constraints are shown in Eq (16).
$$\left\{ \begin{array}{l} \sum_{i}\left((l_{i}f_{i}+w_{i}(1-f_{i}))+G_{1}\right)\leq C_{z_{1}}\\ \sum_{i}\left((l_{i}f_{i}+w_{i}(1-f_{i}))+G_{1}\right)\leq C_{z_{2}}\\ \sum_{i}\left((l_{i}(1-f_{i})+w_{i}f_{i})+G_{1}\right)\leq K_{z_{3}}\\ \sum_{i}\left((l_{i}(1-f_{i})+w_{i}f_{i})+G_{1}\right)\leq K_{z_{4}} \end{array}\right.$$ (16)
  $C_{z_{1}}$ and $C_{z_{2}}$ represent the line wrapping constraints of Z1, Z2 planes, in many cases $C_{z_{1}}$ = $C_{z_{2}}$.
  $K_{z_{3}}$ and $K_{z_{4}}$ represents the line wrapping constraints of Z3 and Z4 planes, in many cases $K_{z_{3}}$ = $K_{z_{4}}$.
  G_**1**_ represents the horizontal device spacing limit.

### Algorithm design

A large number of scholars use NSGA II to solve the equipment layout problem of manufacturing cells [30–32]. The main reason is that the NSGA II algorithm is mature, efficient, and stable in operation. Especially for equipment layout problems, NSGA II is easy to implement and adjust. Because the number of objectives of the equipment layout problem of traditional SCMC is mainly less than three, scholars have been very mature in using NSGA II to solve this problem. Professor Deb has confirmed that NSGA II is not effective in solving problems with more than three objectives, while NSGA III is better for solving more than three objectives. Although NSGA III is less used to solve the equipment layout of manufacturing cells, it has been applied to other industries. For example, Opris A [33], Tang H [34], Sethi K C [35], etc. Because MCMC has five objective functions, this article uses NSGA III to solve this problem and explores the advantages and disadvantages of the NSGA III for solving MCMC.

*(1)*Coding and population initialization

The equipment layout problem is a combinatorial optimization problem of equipment arrangement. Symbolic encoding is commonly used, so this article also uses symbolic encoding.

The equipment codes of MCMC represent the equipment codes of four plane layouts: Z1, Z3, Z2, and Z4. The coding sequence is Z1, Z4, and the equipment layout sequence is from right to left. The equipment layout sequence in Z2 and Z3 planes is from left to right. The equipment layout layers are arranged from the bottom to the top. The coding plane sequence of MCMC is [Z1, Z3, Z2, Z4]. As shown in Fig 4, the MCMC encoding result is [1–15].

From Fig 4, it is easy to find that the equipment center coordinate values in the four planes Z1, Z3, Z2, and Z4 in the multi-layer manufacturing cell have certain characteristics. For example, the z value in the Z1 plane is 0, the x value in the Z3 plane is 0, the x value in the Z2 plane is a fixed value, and the x value in the Z4 plane is a fixed value. For the convenience of decoding after encoding, the coordinate value needs to be followed by the symbol encoding. For example [1(*x*_1_,*y*_1_,*z*_1_),2(*x*_2_,*y*_2_,*z*_2_),…,*n*(*x*_*n*_,*y*_*n*_,*z*_*n*_)], the characteristics of the coordinate values directly represent the locations of the four planes of Z1, Z3, Z2, and Z4 where the equipment is located.

In the evolutionary algorithm of equipment layout, many scholars use random population initialization for the initial population. Therefore, this paper also uses the initial layout population of randomly generated MCMC as the initial population.

*(2)*Crossover and mutation

**Fig 8:**
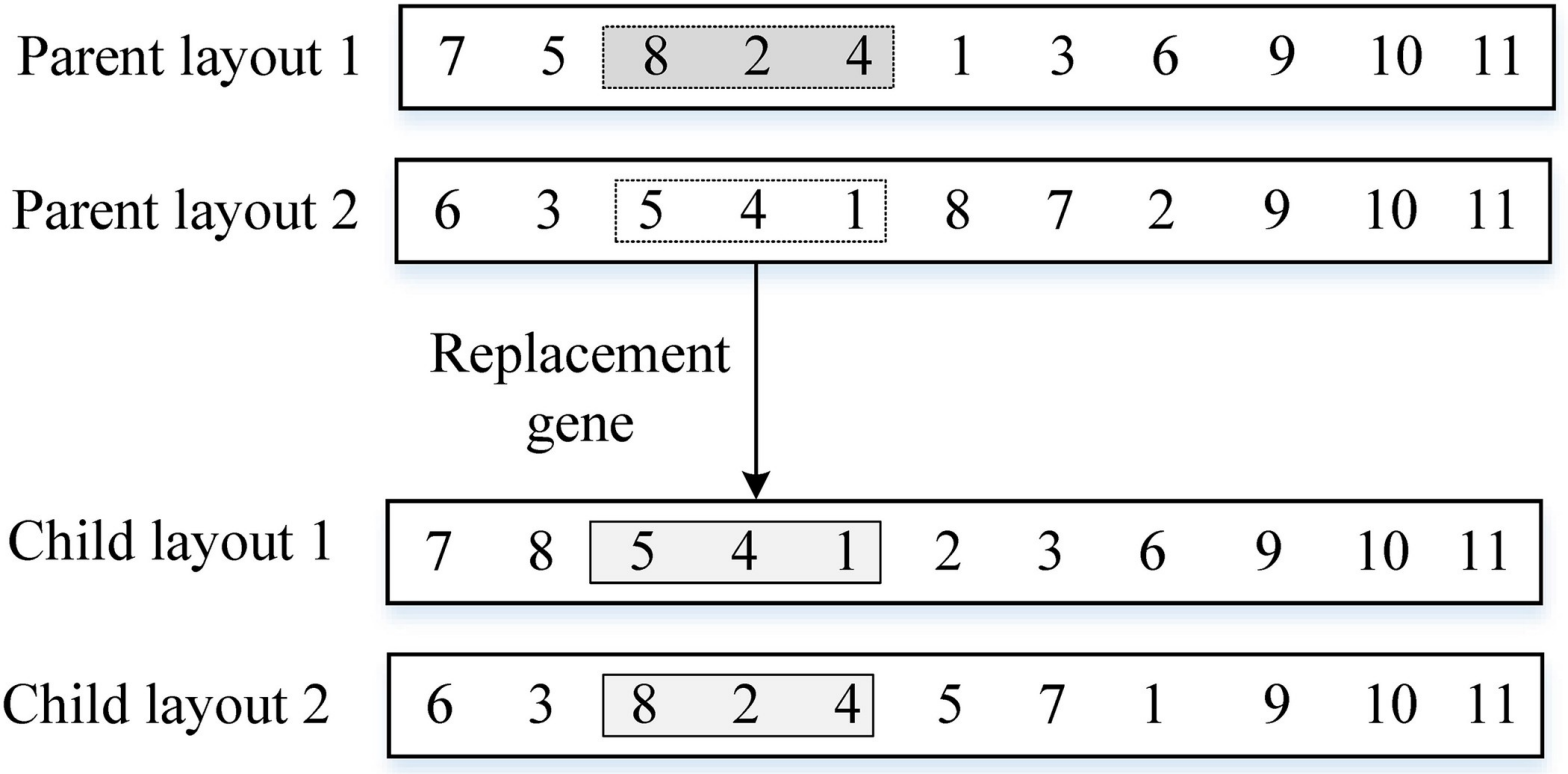
MCMC’s equipment layout cross operation.

The crossover operation of parent individuals is the primary way for NSGA III to generate new individuals and form new populations. It involves exchanging gene segments between parent individuals according to the crossover probability so that excellent genes can be continuously inherited. MCMC’s equipment layout adopts symbolic sequence encoding, intercepts the gene segments of the layout individual based on two randomly selected points, and exchanges the gene segments to form a new layout individual, as shown in Fig 8.

Mutation operation simulates genetic mutations in biological evolution. It can prevent the population from falling in-to the local optimal solution to a certain extent and increase the diversity of the population.

**Fig 9:**
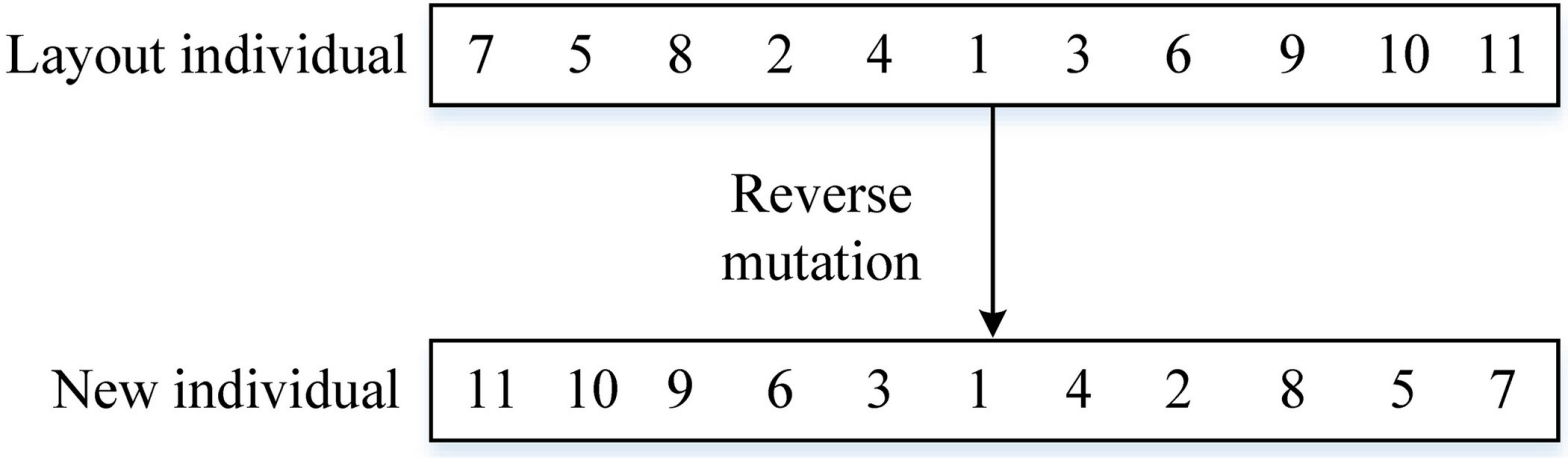
MCMC’s equipment layout mutation operation.

The use of reverse sequence mutation for sequentially coded individuals is highly convenient and operable. The MCMC equipment layout reverse sequence mutation operation is shown in Fig 9.

*(3)*Population elite retention strategy

**Fig 10:**
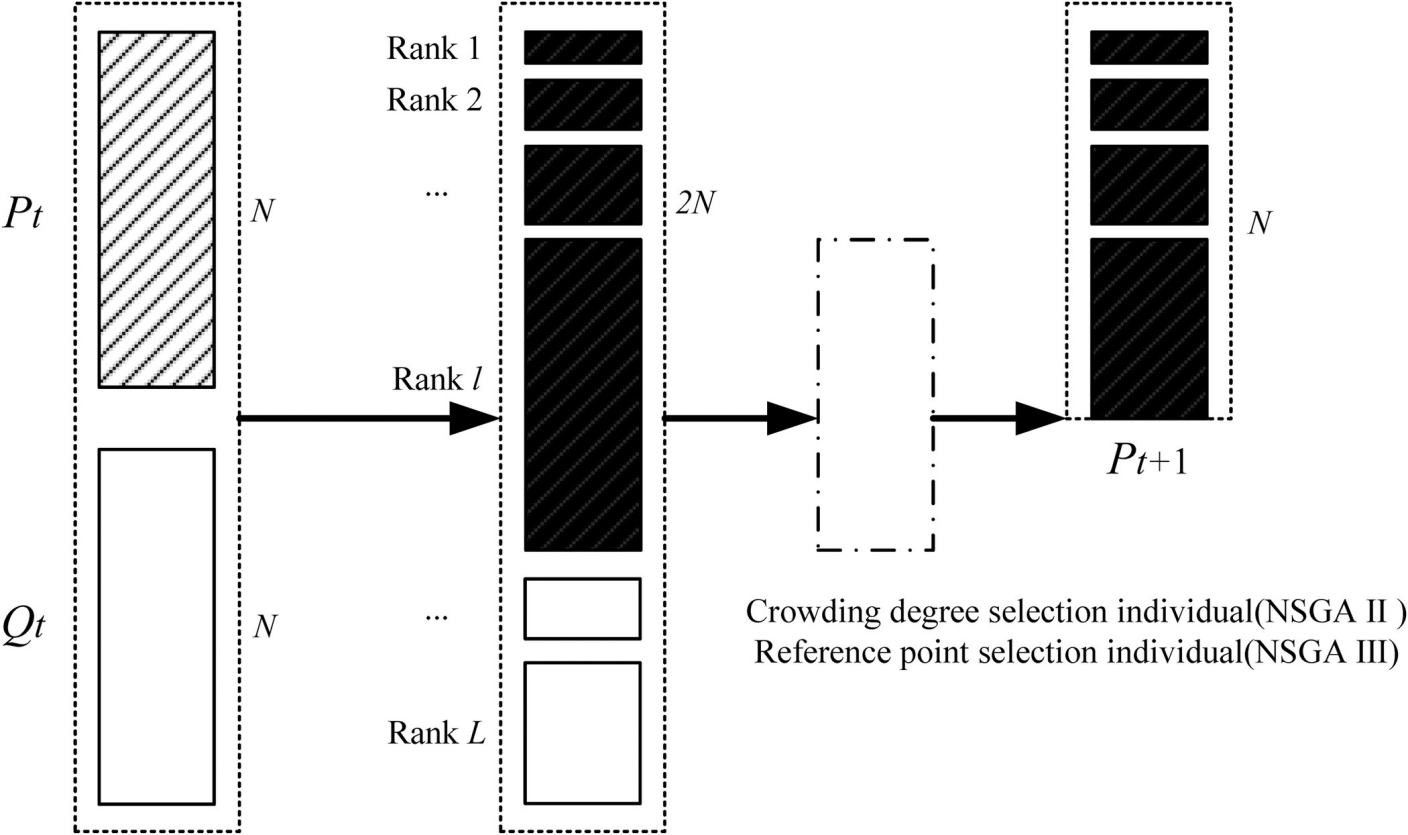
MCMC’s elite retention strategy.

The elite retention strategy is a key step to ensure the population convergence of MCMC. It is also a strategy used by both the fast non-dominated sorting genetic algorithm and the reference point-based non-dominated genetic algorithm. The elite retention strategy is shown in Fig 10.

As shown in Fig 10, the parent population is assumed to be *P*_*t*_ and the offspring population is *Q*_*t*_. The parent and offspring population are merged into the [*P*_*t*_, *Q*_*t*_] population, and the number of individuals in the new population is 2*N*. Perform non-dominated sorting on the new population [*P*_*t*_, *Q*_*t*_], and the sorting result is [Rank 1, Rank 2, Rank 3,…, Rank *L*] layer, where Rank *l* is a column vector. If the total number of individuals from Rank 1 to Rank *l* is equal to *N*, then select these *N* individuals as the next generation population *P*_*t*+1_. If the total number of individuals from Rank 1 to Rank (*l*-1) is less than *N*, and the total number of individuals from Rank 1 to Rank *l* is greater than *N*, then individuals in Rank *l* need to be selected. Using crowding degree selection is NSGA Ⅱ, and using reference point selection is NSGA Ⅲ.

For information on the crowding degree calculation of NSGA Ⅱ and the reference point calculation of NSGA Ⅲ, please refer to Deb[21,22,27].

*(4)*Algorithmic process

**Fig 11:**
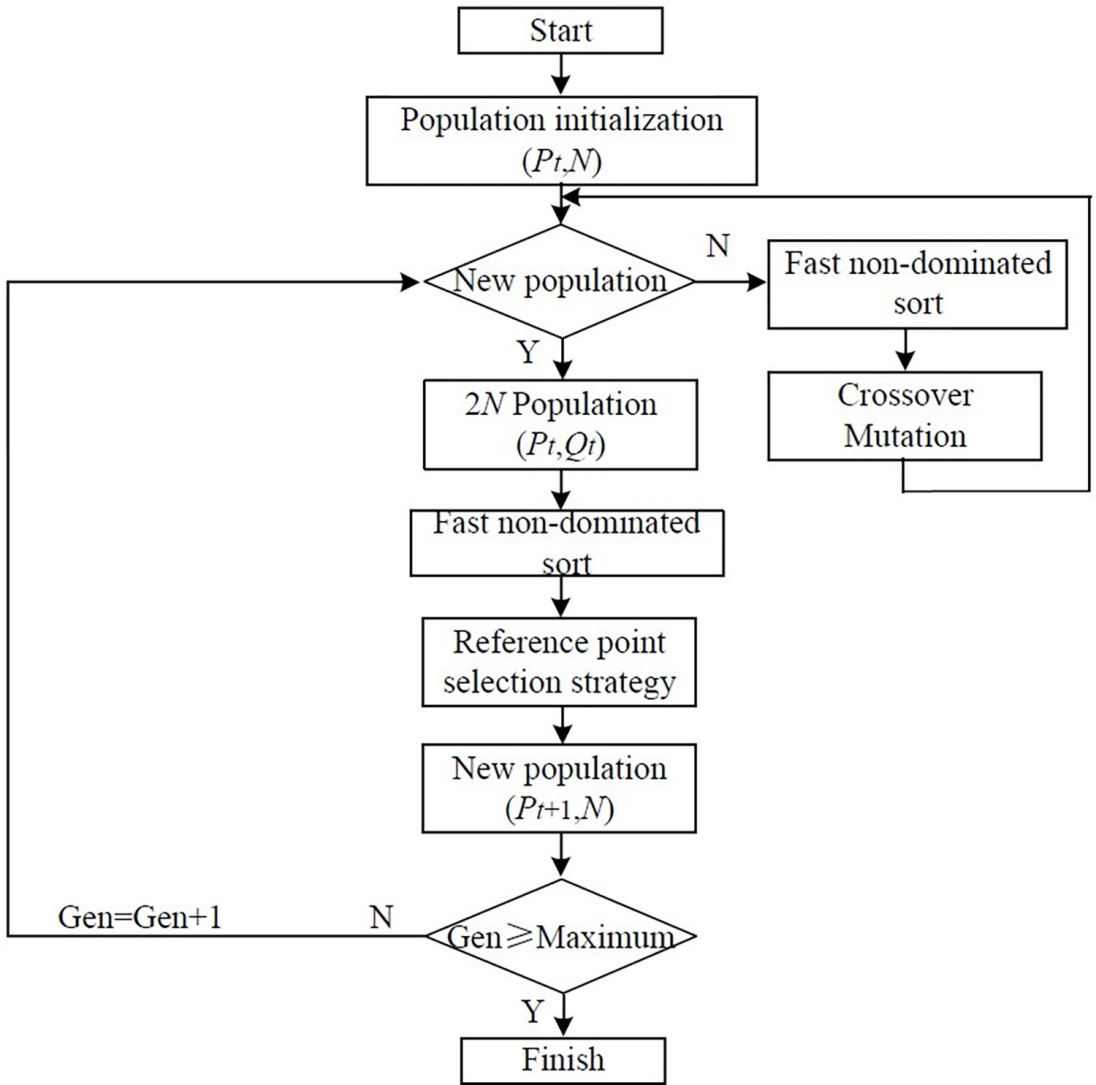
NSGA Ⅲ algorithm.

Fig 11 shows the main process of NSGA III solving the equipment layout problem of MCMC.

NSGA III and NSGA II have the same algorithm structure. They will have fast non-dominated sorting and population crossover and mutation. The only difference is the strategy for selecting the 2*N* population. NSGA II uses the crowding strategy, and NSGA III uses the reference point. The algorithm pseudocode of NSGA III is as follows.

step 1: *S*_*t*_ = *∅*,*i* = 1

step 2: *Q*_*t*_ = *cross*(*P*_*t*_)+mutation(*P*_*t*_)

step 3: *R*_*t*_ = *P*_*t*_∪*Q*_*t*_

step 4: (Rank 1,Rank2,…) = *Non dominated sort* (*R*_*t*_)

step 5: repeat:

*S*_*t*_ = *S*_*t*_∪Rank *i i*=*i*+1

until length(*S*_*t*_)≥*N*

step 6: Last front to be included: Rank *l* = Rank *i*

step 7: if length(*S*_*t*_) = *N*

*P*_*t*+1_ = *S*_*t*_, break

else

$P_{t+1}=\cup_{i=1}^{l}\mathrm{Rank}\;i$

Calculate Rank l individuals based on reference points, select *K* individuals

*P*_*t*+1_ = *P*_*t*+1_∪*K*

end if

## 5. Case application

### Case data

**Fig 12:**
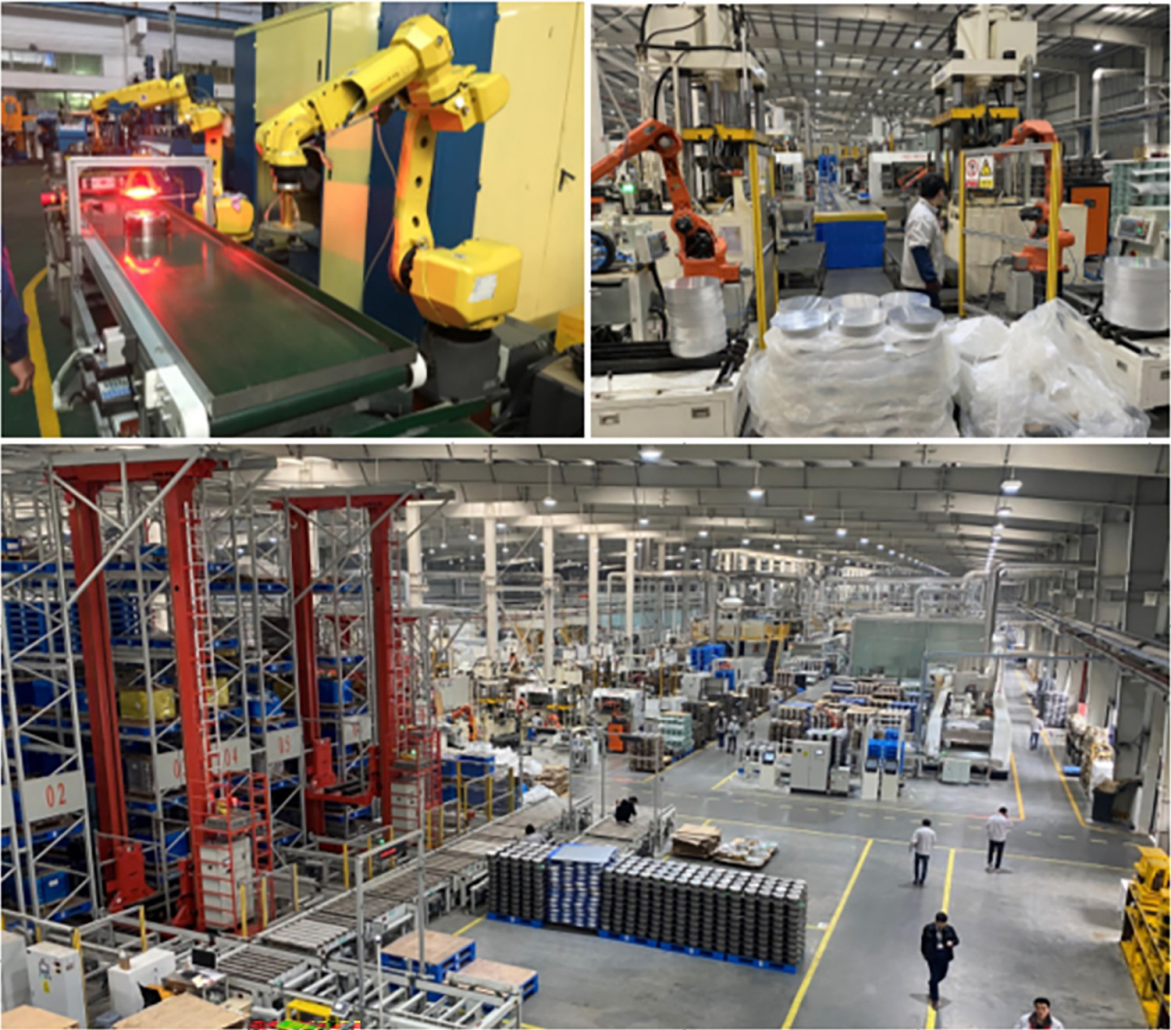
Real scene of case workshop.

This article takes the intelligent workshop of a hardware processing company in Zhejiang as the research object. This workshop mainly produces domestic and foreign kitchen hardware products. The non-stick pans and pressure cookers produced are famous Chinese brand products. The actual scene of the workshop is shown in Fig 12. The products produced in this workshop mainly include small kitchen utensils such as woks, milk soup pots, and frying pans. The main processes include repeating, extrusion, punching, stretching, and diecasting. In engineering applications, the equipment layout is usually between 4 and 10 cells, while academic research on verifying algorithm solutions is mainly between 6 and 15 cells. In the absence of constraints, the scale of the equipment arrangement is the factorial of the number of equipment, and the factorial of 15 equipment is a large-scale optimization problem. In order to verify the effectiveness of the algorithm, this paper selects 17 pieces of equipment in the workshop as the research objects, and codes the equipment. The information of the equipment is shown in Table 4.

**Table 4:** Non-logistics relationship adjacency factor.

*I*	[l_i_,w_i_,h_i_]	W_i_	T_p_
0	[0.50,0.60,0.80]	60	20
1	[0.90,0.80,0.90]	90	15
2	[0.90,0.80,0.110]	200	25
3	[0.70,0.90,1.10]	300	30
4	[0.50,0.60,0.80]	80	12
5	[1.00,0.90,1.00]	100	16
6	[1.70,1.00,1.20]	500	19
7	[0.50,0.60,1.10]	200	29
8	[0.45,0.50,0.90]	60	14
9	[0.40,0.50,0.80]	50	20
10	[1.00,0.90,1.20]	300	18
11	[0.40,0.60,0.80]	70	17
12	[0.70,0.90,1.10]	200	31
13	[0.40,0.70,0.90]	80	24
14	[0.40,0.70,0.80]	50	23
15	[0.50,0.80,1.00]	100	16
16	[0.80,1.00,1.20]	300	15

MCMC has a larger number of equipment, can process more types of products, and has more complex process routes than traditional manufacturing cells. Number the processed products p in the workshop. The processed product information of this workshop is shown in Table 5.

**Table 5:** Device Information.

*p*	Process path	V_p_	V_p_
1	12-2-5-15-4-11-4	5000	10
2	13-9-8-10-7-0-3	6000	10
3	16-3-0-1-7-8-10-13	4000	20
4	5-15-6-14-11-4	5000	20
5	6-10-7-12-11-8-2-0	3000	15

In addition to considering the logistics intensity relationship between equipment and equipment, there are also non-logistics relationships to consider. The importance of non-logistics relationships is represented by five levels: A, E, I, O, and U, as shown in Table 6.

**Table 6:** Non-logistics hierarchy.

*i*	0	1	2	3	4	5	6	7	8	9	10	11	12	13	14	15	16
**0**	-	U	U	U	I	U	U	U	U	U	U	U	U	U	U	U	U
**1**		-	E	I	O	U	A	I	U	U	O	U	U	U	E	U	U
**2**			-	U	U	A	U	U	U	I	U	U	U	U	O	U	U
**3**				-	A	U	I	U	U	U	E	U	U	A	U	U	U
**4**					-	U	U	E	U	E	U	U	U	U	U	U	U
**5**						-	U	U	U	U	U	U	A	U	U	I	U
**6**							-	U	U	O	U	U	U	I	U	U	A
**7**								-	U	U	U	U	U	I	U	U	U
**8**									-	U	U	A	U	U	U	O	U
**9**										-	A	U	U	U	U	U	U
**10**											-	U	U	U	A	I	U
**11**												-	U	U	U	U	I
**12**													-	U	U	A	U
**13**														-	U	U	U
**14**															-	U	O
**15**																-	U
**16**																	-

In addition to the relevant information in the above table, it also includes *δ* = 3, *G*_1_ = 0.2, *G*_2_ = 0.2, *G*_3_ = 1, *C*_z1_ = 2, *C*^z2^ = 2, *K*_z3_ = 2, *K*_z4_ = 2, *f*_*i*_ = 1.

### Case solution

The algorithm population size is 100, the maximum number of iterations is 3000 generations, and each is run 10 times. The operating environment is shown in Table 7.

**Table 7:** Operating environment.

Name	Version/Model	Name	Version/Model
Operating system	Windows 10 Home Chinese Edition	Computer graphics	NVIDIA Gefore GTX1660 Ti
System type	64-bit operating system	Python module	Anaconda3(Python3.7)
Computer processor	Intel(R) Croe(TM) i7-9750H 12 Core	Python module	Numpy
RAM	24 G	Python module	Matplotlib
ROM	2048 G		

**Fig 13:**
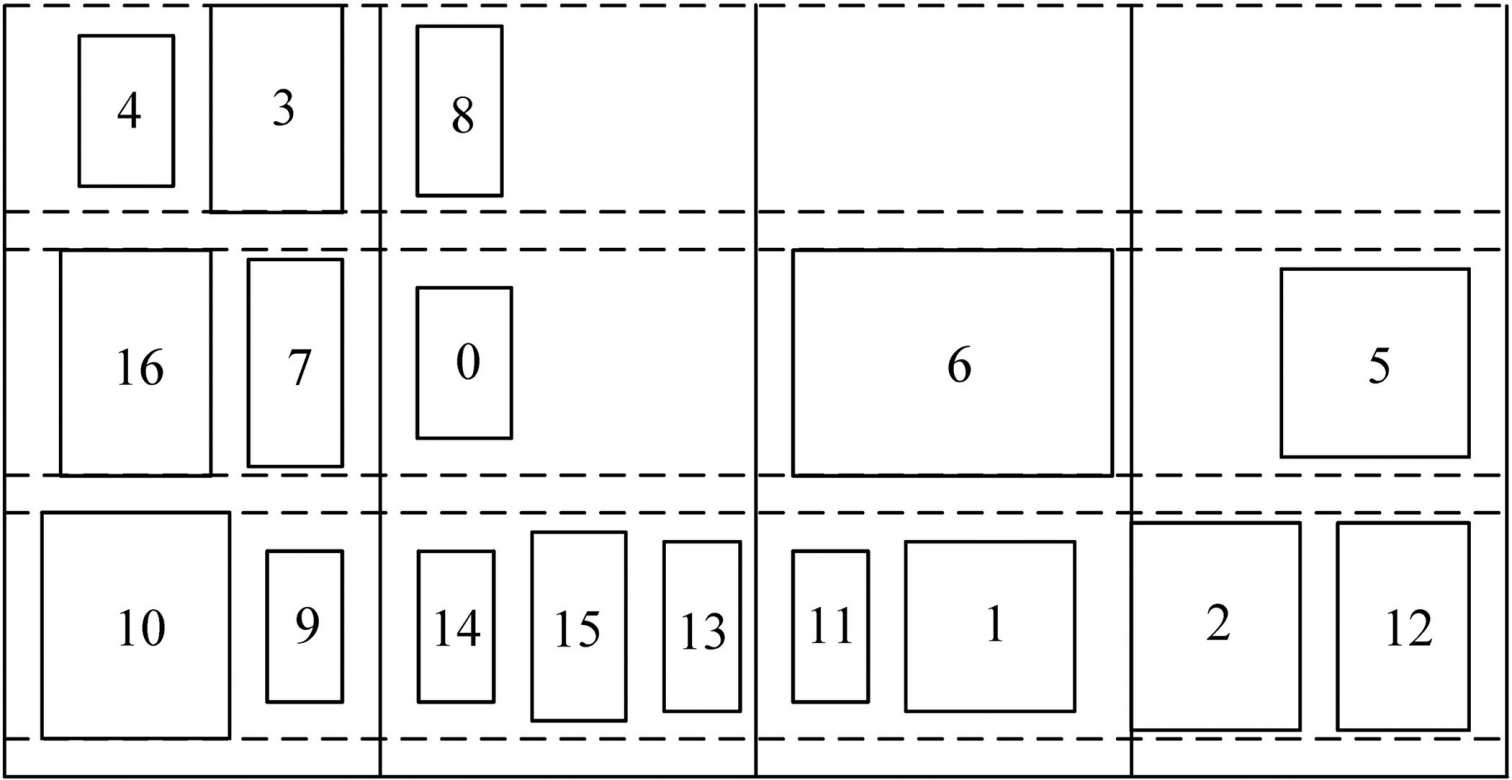
Optimal solution decoding layout.

**Fig 14:**
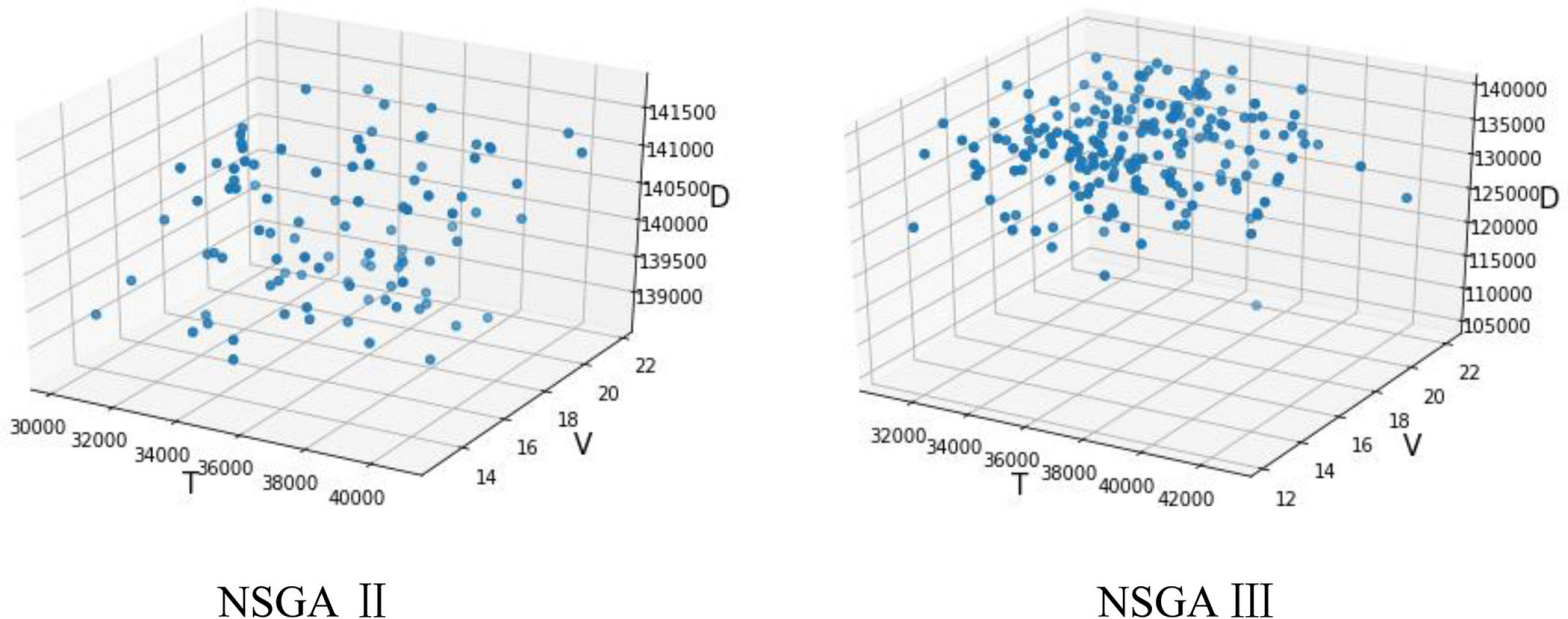
The population individuals of the optimal solution set.

The equipment layout problem of MCMC is simulated and solved using NSGA Ⅱ and NSGA Ⅲ algorithms. Three optimal Pareto solution sets are obtained through the Anaconda3 (Python3.7) program. The Pareto solution set includes coding order, coordinate values, and five target values of *D* (distance), *V* (volume), *T* (loss time), *B* (stability), and *E* (non-logistics) as shown in Table 8. Fig 13 shows the optimal layout decoding obtained by part of MCMC. Fig 14 shows the individual collection of D, V, and T populations in the three layout forms during the calculation process and in the solution process of the two algorithms.

**Table 8:** Example solution results.

Algrithm	Solution results	*D*, *V*, *T*, *B*, *E*
NSGA Ⅱ	16(0.6,0.8,0.0),1(0.0,0.8,0.6),11(0.0,0.8,1.5),3(0.55,0.8,1.95),10(2.35,0.8,0.65),5(0.7,2.2,0.0),6(0.0,2.2,0.7),12(0.55,2.2,1.95),2(1.55,2.2,1.95),4(2.35,2.2,0.5),9(2.35,2.2,1.25),14(2.35,2.2,2.05),13(0.4,3.55,0.0),7(1.1,3.55,0.0),8(0.0,3.55,0.45),15(0.0,3.55,1.3),0(0.45,3.55,1.95)	40037.5,18.7,140985.0,5991.5,17.3
NSGA Ⅱ	8(0.425,0.75,0.0),7(1.15,0.75,0.0),2(0.0,0.75,0.6),1(0.65,0.75,1.95),4(1.55,0.75,1.95),13(2.3,0.75,0.55),14(2.3,0.75,1.45),16(0.6,2.1,0.0),0(1.45,2.1,0.0),12(0.0,2.1,0.65),5(0.7,2.1,1.95),6(2.3,2.1,0.7),15(0.45,3.5,0.0),11(1.1,3.5,0.0),9(1.7,3.5,0.0),3(0.0,3.5,0.65),10,(0.7,3.5,1.95)	40760.0,20.7,141132.5,4980.5,15.7
NSGA Ⅲ	9(0.4,0.8,0.0),10(1.3,0.8,0.0),14(0.0,0.8,0.55),15(0.0,0.8,1.5),13(0.0,0.8,2.45),11(0.4,0.8,2.0),1(1.25,0.8,2.0),12(1.75,0.8,0.65),2(1.75,0.8,1.7),7(0.5,2.2,0.0),16(1.4,2.2,0.0),0(0.0,2.2,0.5),6(1.05,2.2,2.0),5(1.75,2.2,0.65),3(0.55,3.55,0.0),4(1.35,3.55,0.0),8(0.0,3.55,0.45)	40037.5,18.7,140985.0,5991.5,17.3
NSGA Ⅲ	2(0.65,0.8,0.0),12(1.65,0.8,0.0),10(0,0.8,0.65),13(0,0.8,1.65),1(0.65,0.8,2.0),9(1.5,0.8,2.0),11(1.85,0.8,0.5),15(1.85,0.8,1.4),16(0.6,2.2,0.0),8(1.425,2.2,0.0),14(0,2.2,0.55),6(1.05,2.2,2.0),0(1.85,2.2,0.5),3(1.85,2.2,1.45), 5(0.7,3.55,0.0),7(1.7,3.55,0.0),4(0,3.55,0.5)	34565.0,14.9,122230.0,4493.5,14.5

As mentioned above, the five objectives of MCMC are D (distance), V (volume), T (loss time), B (stability), and E (non-logistics), and the five objective values are the quantitative results of the MCMC layout problem. As shown in Table 7, the optimal result [D, V, T, B, E] of NSAG II is [40037.5, 18.7, 140985.0, 5991.5, 17.3], and the optimal result [D, V, T, B, E] of NSGA III is [34565.0, 14.9, 122230.0, 4493.5, 14.5]. This paper compares the objective values of the solution results of NSAG II and NSGA III, as shown in Fig 14.

**Fig 15:**
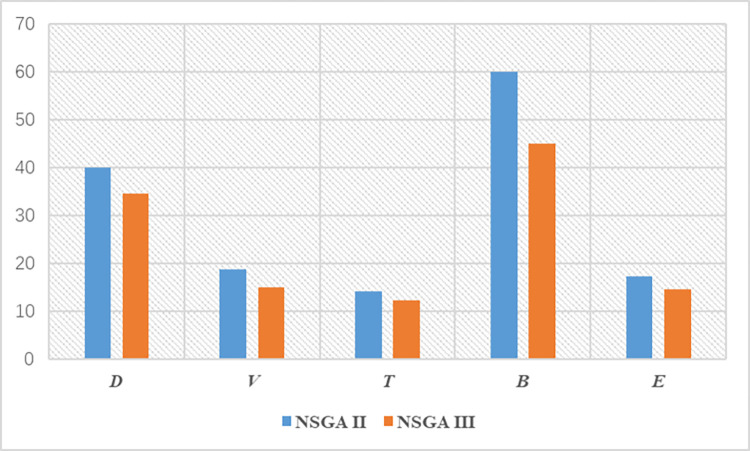
Comparison of target values solved by NSAG II and NSGA III (unit: *D*(10^3^), *V*(10), *T*(10^4^), *B*(10^2^), *E*(10)).

As shown in Fig 15, the yellow bar graph represents the solution results of NSGA III, and the blue bar graph represents the solution results of NSAG II. In the five-dimensional target [D, V, T, B, E], the solution results of NSGA III are better than those of NSAG II. The results show that the results of NSGA III in solving the MCMC equipment layout problem with high-dimensional targets are better than those of NSAG II.

The solution results show that NSGA III is significantly better than NSAG II. As the number of targets increases, there are more individuals at the same level (Rank *i*). When individuals are selected in the key layer (Rank *l*), the population diversity of the NSAG II selection method using crowding degree is significantly lower than that of NSGA III using reference point selection. The population diversity of NSAG II and NSGA III is shown in Fig 14. Through the above case analysis, it can be concluded that NSGA III based on the reference point performs well in solving multi-objective MCMC and has strong superiority.

The application of this case describes the application and solution of MCMC of multi-dimensional objective functions. Also, it verifies the effectiveness of NSGA III in solving high-dimensional MCMC equipment layout. The model has a certain degree of versatility. For example, the number of targets can be freely increased or decreased, and the constraints can also be changed. For instance, in a manufacturing enterprise where the equipment weight is not large, the stability target and constraints can be deleted. For example, in a semi-automated manufacturing enterprise, human-machine cooperation is required, and human-machine cooperation targets and constraints need to be added. In addition to the above examples, many enterprises can use NSGA III to solve the model by increasing or decreasing targets and constraints. In summary, the model has a certain degree of versatility.

## 6. Managerial insights

The findings of this study carry several important implications for managers in smart manufacturing environments. Firstly, the introduction of Multi-layer Circular Manufacturing Cells (MCMC) presents an opportunity for enhanced operational efficiency and flexibility. Managers should consider implementing MCMC layouts to optimize material handling and reduce operational bottlenecks, ultimately leading to improved production throughput.

Moreover, the application of the NSGA III algorithm demonstrates the value of advanced analytical tools in addressing complex layout challenges. Managers are encouraged to invest in algorithmic solutions and training to equip their teams with the necessary skills to leverage these technologies effectively. By adopting innovative layout strategies and analytical methods, organizations can remain competitive in a rapidly evolving manufacturing landscape.

Furthermore, the multi-objective model developed in this study provides a robust framework for decision-making. Managers can utilize this model to evaluate trade-offs among various factors such as cost, space utilization, and equipment interactions. This holistic approach enables informed decisions that align with organizational goals and resource constraints.

Finally, as the industry transitions toward more integrated manufacturing systems, managers should be proactive in exploring the synergy between single-layer and multi-layer manufacturing cells. This integration can lead to greater adaptability and responsiveness to market demands, enhancing overall operational resilience.

## 7. Conclusions, limitations, and recommendations for future research

### Findings

This paper addresses the engineering demand for Multi-layer Circular Manufacturing Cells (MCMC) in smart factory construction by proposing a comprehensive layout method and establishing a multi-objective mathematical model. The NSGA III algorithm was designed to effectively solve the MCMC layout problem, demonstrating superior performance compared to NSGA II. Key contributions include pioneering research on MCMC layouts, providing a foundational framework for future studies, and establishing a complex multi-objective model that incorporates various factors such as material handling, volume, loss time, stability, and non-logistics relationships. Additionally, the constraints considered in this study enhance the applicability of the proposed model.

### Research limitations

While this research offers significant insights, it is important to acknowledge certain limitations. The case studies presented may not fully represent all potential scenarios in smart factory environments, as variations in manufacturing processes and equipment types could influence layout efficiency. Furthermore, the complexity of the mathematical model may pose challenges for practical implementation in diverse industrial contexts.

### Recommendations for future research

Future research should focus on several areas to build upon this study. First, exploring real-time solutions for equipment layout problems using AR/VR technology in digital factories represents a promising direction. Machine learning can be leveraged for real-time adjustments, requiring extensive data for effective model training. Second, integrating the application of single-layer and multi-layer manufacturing cells within factories could yield valuable insights, albeit with increased complexity and challenges. Addressing these future challenges will enhance the adaptability and efficiency of manufacturing layouts in evolving smart factory environments.

## Supporting information

S1 Data (ZIP)
