# Supplementary material for: Research on equipment layout of multi-layer circular manufacturing cell based on NSGA III
Source: PLoS One. 2024 Dec 23;19(12):e0312364. doi: 10.1371/journal.pone.0312364 (PMC11666037; doi:10.1371/journal.pone.0312364)
Supplement: S1 Data — (ZIP) [file pone.0312364.s001.zip › Environmental parameters and result data.docx]

**1.** **Operating environment**

| **Name** | **Version/Model** | **Name** | **Version/Model** |
| --- | --- | --- | --- |
| Operating system | Windows 10 Home Chinese Edition | Computer graphics | NVIDIA  Gefore GTX1660 Ti |
| System type | 64-bit operating system | Python module | Anaconda3(Python3.7) |
| Computer processor | Intel(R)  Croe(TM) i7-9750H 12 Core | Python module | Numpy |
| RAM | 24 G | Python module | Matplotlib |
| ROM | 2048 G |  |  |

**2. Solution results**

| Algrithm | Solution results | *D*, *V*, *T*, *B*, *E* |
| --- | --- | --- |
| NSGA Ⅱ | 16(0.6,0.8,0.0),1(0.0,0.8,0.6),11(0.0,0.8,1.5),3(0.55,0.8,1.95),10(2.35,0.8,0.65),5(0.7,2.2,0.0),6(0.0,2.2,0.7),12(0.55,2.2,1.95),2(1.55,2.2,1.95),4(2.35,2.2,0.5),9(2.35,2.2,1.25),14(2.35,2.2,2.05),13(0.4,3.55,0.0),7(1.1,3.55,0.0),8(0.0,3.55,0.45),15(0.0,3.55,1.3),0(0.45,3.55,1.95) | 40037.5,18.7,140985.0,5991.5,17.3 |
| NSGA Ⅱ | 8(0.425,0.75,0.0),7(1.15,0.75,0.0),2(0.0,0.75,0.6),1(0.65,0.75,1.95),4(1.55,0.75,1.95),13(2.3,0.75,0.55),14(2.3,0.75,1.45),16(0.6,2.1,0.0),0(1.45,2.1,0.0),12(0.0,2.1,0.65),5(0.7,2.1,1.95),6(2.3,2.1,0.7),15(0.45,3.5,0.0),11(1.1,3.5,0.0),9(1.7,3.5,0.0),3(0.0,3.5,0.65),10,(0.7,3.5,1.95) | 40760.0,20.7,141132.5,4980.5,15.7 |
| NSGA Ⅲ | 9(0.4,0.8,0.0),10(1.3,0.8,0.0),14(0.0,0.8,0.55),15(0.0,0.8,1.5),13(0.0,0.8,2.45),11(0.4,0.8,2.0),1(1.25,0.8,2.0),12(1.75,0.8,0.65),2(1.75,0.8,1.7),7(0.5,2.2,0.0),16(1.4,2.2,0.0),0(0.0,2.2,0.5),6(1.05,2.2,2.0),5(1.75,2.2,0.65),3(0.55,3.55,0.0),4(1.35,3.55,0.0),8(0.0,3.55,0.45) | 40037.5,18.7,140985.0,5991.5,17.3 |
| NSGA Ⅲ | 2(0.65,0.8,0.0),12(1.65,0.8,0.0),10(0,0.8,0.65),13(0,0.8,1.65),1(0.65,0.8,2.0),9(1.5,0.8,2.0),11(1.85,0.8,0.5),15(1.85,0.8,1.4),16(0.6,2.2,0.0),8(1.425,2.2,0.0),14(0,2.2,0.55),6(1.05,2.2,2.0),0(1.85,2.2,0.5),3(1.85,2.2,1.45),  5(0.7,3.55,0.0),7(1.7,3.55,0.0),4(0,3.55,0.5) | 34565.0,14.9,122230.0,4493.5,14.5 |

**3. Algorithm pseudocode**

step 1: $S_{t}=\emptyset,i=1$

step 2: $Q_{t}=cross\left(P_{t}\right)+\text{mutation}\left(P_{t}\right)$

step 3: $R_{t}=P_{t}\cup Q_{t}$

step 4: $\left(\text{Rank 1,Rank 2,}\cdots\right)\text{=}Non\text{ }dominated\text{ }sort\left(R_{t}\right)$

step 5: repeat:

$$S_{t}=S_{t}\cup\text{Rank }i\begin{matrix} & i=i+1 \end{matrix}$$

until length(*S_t_*)≥*N*

step 6: Last front to be included: Rank *l*= Rank *i*

step 7: if length(*S_t_*)=*N*

$P_{t+1}=S_{t}$, break

else

$$P_{t+1}=\cup_{i=1}^{l}\text{Rank }i$$

Calculate Rank l individuals based on reference points, select *K* individuals

$$P_{t+1}=P_{t+1}\cup K$$

end if
