# Supplementary material for: Research on equipment layout of multi-layer circular manufacturing cell based on NSGA III
Source: PLoS One. 2024 Dec 23;19(12):e0312364. doi: 10.1371/journal.pone.0312364 (PMC11666037; doi:10.1371/journal.pone.0312364)
Supplement: S1 Data — (ZIP) [file pone.0312364.s001.zip › Enterprise Data.docx]

**1. Non-logistics relationship adjacency factor**

| ***i*** | [l_i_,w_i_,h_i_] | W_i_ | T_p_ |
| --- | --- | --- | --- |
| 0 | [0.50,0.60,0.80] | 60 | 20 |
| 1 | [0.90,0.80,0.90] | 90 | 15 |
| 2 | [0.90,0.80,0.110] | 200 | 25 |
| 3 | [0.70,0.90,1.10] | 300 | 30 |
| 4 | [0.50,0.60,0.80] | 80 | 12 |
| 5 | [1.00,0.90,1.00] | 100 | 16 |
| 6 | [1.70,1.00,1.20] | 500 | 19 |
| 7 | [0.50,0.60,1.10] | 200 | 29 |
| 8 | [0.45,0.50,0.90] | 60 | 14 |
| 9 | [0.40,0.50,0.80] | 50 | 20 |
| 10 | [1.00,0.90,1.20] | 300 | 18 |
| 11 | [0.40,0.60,0.80] | 70 | 17 |
| 12 | [0.70,0.90,1.10] | 200 | 31 |
| 13 | [0.40,0.70,0.90] | 80 | 24 |
| 14 | [0.40,0.70,0.80] | 50 | 23 |
| 15 | [0.50,0.80,1.00] | 100 | 16 |
| 16 | [0.80,1.00,1.20] | 300 | 15 |

2. Device Information

| ***p*** | Process path | V_p_ | V_p_ |
| --- | --- | --- | --- |
| 1 | 12-2-5-15-4-11-4 | 5000 | 10 |
| 2 | 13-9-8-10-7-0-3 | 6000 | 10 |
| 3 | 16-3-0-1-7-8-10-13 | 4000 | 20 |
| 4 | 5-15-6-14-11-4 | 5000 | 20 |
| 5 | 6-10-7-12-11-8-2-0 | 3000 | 15 |

3. Non-logistics hierarchy

| ***i*** | **0** | **1** | **2** | **3** | **4** | **5** | **6** | **7** | **8** | **9** | **10** | **11** | **12** | **13** | **14** | **15** | **16** |
| --- | --- | --- | --- | --- | --- | --- | --- | --- | --- | --- | --- | --- | --- | --- | --- | --- | --- |
| **0** | - | U | U | U | I | U | U | U | U | U | U | U | U | U | U | U | U |
| **1** |  | - | E | I | O | U | A | I | U | U | O | U | U | U | E | U | U |
| **2** |  |  | - | U | U | A | U | U | U | I | U | U | U | U | O | U | U |
| **3** |  |  |  | - | A | U | I | U | U | U | E | U | U | A | U | U | U |
| **4** |  |  |  |  | - | U | U | E | U | E | U | U | U | U | U | U | U |
| **5** |  |  |  |  |  | - | U | U | U | U | U | U | A | U | U | I | U |
| **6** |  |  |  |  |  |  | - | U | U | O | U | U | U | I | U | U | A |
| **7** |  |  |  |  |  |  |  | - | U | U | U | U | U | I | U | U | U |
| **8** |  |  |  |  |  |  |  |  | - | U | U | A | U | U | U | O | U |
| **9** |  |  |  |  |  |  |  |  |  | - | A | U | U | U | U | U | U |
| **10** |  |  |  |  |  |  |  |  |  |  | - | U | U | U | A | I | U |
| **11** |  |  |  |  |  |  |  |  |  |  |  | - | U | U | U | U | I |
| **12** |  |  |  |  |  |  |  |  |  |  |  |  | - | U | U | A | U |
| **13** |  |  |  |  |  |  |  |  |  |  |  |  |  | - | U | U | U |
| **14** |  |  |  |  |  |  |  |  |  |  |  |  |  |  | - | U | O |
| **15** |  |  |  |  |  |  |  |  |  |  |  |  |  |  |  | - | U |
| **16** |  |  |  |  |  |  |  |  |  |  |  |  |  |  |  |  | - |
